# Authorship Trends of Emergency Medicine Publications over the Last Two Decades

**DOI:** 10.5811/westjem.2016.2.29779

**Published:** 2016-05-05

**Authors:** Richard Lammers, Thomas Simunich, John Ashurst

**Affiliations:** * Duke Lifepoint Conemaugh Memorial Medical Center, Department of Research, Johnstown, Pennsylvania; † Duke Lifepoint Conemaugh Memorial Medical Center, Department of Emergency Medicine, Johnstown, Pennsylvania

## Abstract

**Introduction:**

With the recent merger of the American Osteopathic Association (AOA) and the Accreditation Council for Graduate Medical Education (ACGME) a heightened pressure for publication may become evident. Our objective was to determine whether there was a gap in the type of both medical degree designation and advanced degree designation among authorship in three United States-based academic emergency medicine journals.

**Methods:**

We reviewed the *Journal of Emergency Medicine*, *Academic Emergency Medicine* and *Annals of Emergency Medicine* for the type of degree designation that the first and senior authors had obtained for the years 1995, 2000, 2005, 2010 and 2014.

**Results:**

A total of 2.48% of all authors held a degree in osteopathic medicine. Osteopathic physician first authors contributed to 3.26% of all publications while osteopathic physician senior authors contributed 1.53%. No statistical trend could be established for the years studied for osteopathic physicians. However, we noted an overall trend for increased publication for allopathic senior authors (p=0.001), allopathic first authors with a dual degree (p=0.003) and allopathic senior authors with a dual degree (p=0.005). For each journal studied, no statistical trend could be established for osteopathic first or senior authors but a trend was noted for allopathic first and senior authors in the *Journal of Emergency Medicine* (p-value=0.020 and 0.006). Of those with dual degrees, osteopathic physicians were in the minority with 1.85% of osteopathic first authors and 0.60% of osteopathic senior authors attaining a dual degree. No statistical trend could be established for increased dual degree publications for osteopathic physicians over the study period, nor could a statistical trend be established for any of the journals studied.

**Conclusion:**

Very few osteopathic physicians have published in the *Journal of Emergency Medicine*, *Academic Emergency Medicine* or *Annals of Emergency Medicine* over the last two decades. Despite a trend for increased publication by allopathic physicians in certain journals, there appears to be no trend for increased publication of osteopathic physicians in emergency medicine.

## INTRODUCTION

With the recent merger of the American Osteopathic Association (AOA) and the Accreditation Council for Graduate Medical Education (ACGME) new standards will be established for scholarly activity criteria and designation for each specialty. The ACGME requires “all core faculty members to be involved in scholarly projects” with “one scientific peer-reviewed publication for every five core faculty members per year.”^1^ The American College of Osteopathic Emergency Physicians (ACOEP) requirements state “scholarly activity should include a minimum of 2 major or 1 major and 2 minor scholarly activities” every four years.^2^ Some of these activities include serving on committee, participating in item writing or grant writing, manuscript publications, or serving as a judge/moderator for an academic meeting.^2^

Recent research has shown that 39% of allopathic emergency medicine (EM) residencies require their residents to participate in an “original research project,” but this research did not include osteopathic residencies or faculty members in these results.^3^ Several other studies have also highlighted that osteopathic EM residencies are under-represented in top tier EM journal publications and very few editors of top tier academic journals are osteopathic physicians.^4^,^5^ In this study, we sought to determine if there was a difference between allopathic and osteopathic physician publications in EM journals over the last two decades.

## METHODS

### Study Design

This was a retrospective study of graduate degree (M.D. vs D.O.) trends in publications from the *Annals of Emergency Medicine* (*Annals*), *Academic Emergency Medicine* (*AEM*), and the *Journal of Emergency Medicine* (*JEM*). We chose journals based upon impact factor and citation half-life. For each author, reviewers designated medical degree based on the names listed with each article. An author’s graduate degree was determined by initial inspection of his or her suffix. For those with more than one advanced degree, both the medical degree and advanced degree were tallied for analysis. In articles published by a single author, the author was considered the first author for statistical purposes. All original articles, review articles, case reports, and image reports were reviewed for the type of medical degree the first and senior author had obtained from 1995, 2000, 2005, 2010 and 2014 by searching each journal’s web portal. We excluded from analysis articles such as media reviews, editorials, book reviews, letters to the editor and correspondences.

### Data Analysis

We obtained comparison of the proportions of allopathic and osteopathic physician authorship across the years by using simple descriptive statistics. The percentage of those holding both a medical degree and an advanced degree was also analyzed using descriptive statistics. We analyzed trends in authorship using simple linear regression.

## RESULTS

We reviewed 3,189 articles and 5,805 authors for the years studied. A total of 3,189 authors were considered first authors and 810 of these authors held a dual degree. A total of 573 manuscripts were authored by a single author as well. A total of 2,616 authors were considered the senior author on a manuscript, with 667 of these authors holding a dual degree.

Overall, 2.48% (144/5805) of authors held a degree in osteopathic medicine. Of those authors considered to be the first author, 3.26% (104/3189) held a degree in osteopathic medicine and 1.53% (40/2616) of senior authors held a degree in osteopathic medicine. Similarly, 1.85% (15/812) of first authors who held a dual advanced degree and 0.60% (4/667) of senior authors who held a dual advanced degree also had a degree in osteopathic medicine. Further analysis into each journal shows that 2.71% (48/1771) of all authors in *JEM*, 2.57% (50/1943) in *AEM* and 2.20% (46/2091) in *Annals* were osteopathic physicians.

In 1995 a total of 3.82% (21/550) of osteopathic physicians served in the first author role and 2.06% (9/436) of osteopathic physicians served in the senior author role, while in 2014 a total of 2.68% (19/708) and 1.46% (9/615) of osteopathic physicians served in this role (Table 1). Likewise, in 1995 a total of 1.27% (1/79) of osteopathic physicians served in the first author role and held an advanced degree as compared to 1.23% (3/246) in 2014 (Table 2). For those holding both an osteopathic degree and an advanced degree who served in the senior author role in 1995, 0% (0/80) were osteopathic physicians as compared to 0.93% (2/214) in 2014 (Table 2).

Overall, no trend was noted for increased osteopathic physician publication in either the first or senior author spot over time (p=0.375 and p=0.882). Neither could we establish a statistically significant trend for first and senior osteopathic physician publication over the years studied in any journal (Table 1). In several journals, however, statistically significant trends were established for allopathic publication for both the senior author and first author over time (Table 1).

## DISCUSSION

This study highlights the significant disparity in degrees held by those publishing in three American academic EM journals. Based upon the results, osteopathic physicians publish less frequently in the journals studied and hold fewer advanced degrees as compared to their allopathic counterparts.

Previous literature has shown that authors affiliated with osteopathic EM residencies are under-represented in high impact academic EM journals, but that study was only conducted for the year 2011.^4^ The static nature of reviewing only a single year made that data difficult to extrapolate. Based upon this study, however, osteopathic EM physicians have been under-represented in several key EM journals over the last two decades. Even as time has progressed, no trend could be established for increased publication by both first and senior authors who hold an osteopathic medical degree.

No study to date has directly explored why osteopathic physicians are less likely to publish manuscripts in journals, but several limitations for those with the osteopathic medical degree may exist that their allopathic counterparts do not face. For instance, allopathic residencies are at large academic institutions with a trend for increased funding, protected academic time, and large collaborative trials.^6^ These added resources may allow allopathic physicians the opportunity to publish articles at an increased rate. Another barrier that may exist is that there is insufficient research training in graduate medical education. After a survey of residents, Riveria et al noted the largest barriers to completing a successful research project were the lack of time, having inadequate research skills and a lack of a formal research curriculum.^7^

According to our results, allopathic physicians who hold a dual degree have been increasingly publishing manuscripts in the EM literature. In 2004, the American Association of Colleges of Osteopathic Medicine noted that only 26 osteopathic medical students were enrolled in a dual DO/PhD degree program, while in 2012 there were only 29.^8^,^9^ Several factors may dissuade students from pursuing a dual DO/PhD degree, among them emphasis on acquiring extramural funding, the length of time to acquire the dual degree and the possibility of decreased income as an academic physician.^10^–^12^ Over the past several years, however, the osteopathic profession has made adjustments to the curriculum to better train dual degreed physicians and now offers 91 combined programs highlighted by nine DO/PhD, 15 DO/MS, and 16 DO/MPH programs.^13^

Since the merger of the AOA and the ACGME, neither organization has commented on an updated research requirement for core faculty. As it stands however, very few core faculty osteopathic EM physicians may meet the requirement if the ACGME standards are adopted. One way to improve the academic output and lay the ground work of scholarly activity for osteopathic EM physicians may be to enroll in the American College of Emergency Physicians Emergency Medicine Basic Research Skills (EMBRS) workshop, the American College of Osteopathic Physicians Faculty Development Workshop, or the Medical Education Research Certification with the Council of Emergency Medicine Program Directors. These programs not only offer the fundamentals of research but also allow for mentoring and collaborative efforts amongst the participants.

## LIMITATIONS

There are several limitations to this study. The authors cannot comment on the years that were not studied, which may have led to other osteopathic physicians who served as either the first or senior author that were not included in the study. The authors also cannot comment on the number of manuscripts submitted to each journal by osteopathic physicians over the time period.

Although unlikely based upon trend analysis, if more or less osteopathic physicians published in these years not surveyed, it may have altered the results. Also, several authors were listed as “unknown” due to no degree being established with their authorship.

While we made every effort to remove all “Letters to the Editor,” during the early years several of the journals did not clearly note these publications as such. All efforts were made to remove these from the study by review of the abstracts when available. Lastly, several journals publish brief reports, case reports and clinical images in the “Letters to the Editor” section. These were not included in the totals due to publication status in the “Letters” section, and it was felt that little change would be present in the results because of this.

## CONCLUSION

Over the last two decades, very few osteopathic physicians published original research articles, review articles, case reports, or image reports in *JEM*, *AEM* and *Annals*. Over the years studied, we saw no trend for increased publication by either the osteopathic first author or osteopathic senior author for the journals studied. As compared to their allopathic counterparts, very few osteopathic first or senior authors hold an advanced degree.

## Figures and Tables

**Table 1:** Percent representation of osteopathic and allopathic physicians among first and senior authors of published articles in three journals of emergency medicine.

	1995	2000	2005	2010	2014	Adj R Sq	P-value
Overall							
DO First	3.82 (21/550)	3.60 (21/583)	3.65 (23/631)	2.79 (20/717)	2.68(19/708)	0.020	0.375
DO Senior	2.06 (9/436)	1.36 (6/442)	1.72 (9/523)	1.17 (7/600)	1.46 (9/615)	−0.322	0.882
MD First	96.18 (529/550)	96.40 (562/583)	96.35 (608/631)	97.21 (697/717)	97.32 (689/708)	0.677	0.055
MD Senior	97.94 (427/436)	98.64 (436/442)	98.28 (514/523)	98.83 (593/600)	98.54 (606/615)	0.978	0.001
JEM							
DO First	3.91 (5/128)	5.63 (8/142)	2.89 (5/173)	3.36 (8/238)	2.85 (7/246)	−0.092	0.475
DO Senior	2.88 (3/104)	2.5 (3/120)	1.85 (3/162)	0.45 (1/223)	2.13 (5/235)	−0.287	0.764
MD First	96.09 (123/128)	94.37 (134/142)	97.11 (168/173)	96.64 (230/238)	97.15 (239/246)	0.833	0.020
MD Senior	97.12 (101/104)	97.5 (117/120)	98.15 (159/162)	99.55 (222/223)	97.87 (230/235)	0.927	0.006
Acad EM							
DO First	3.24 (6/185)	3.40 (8/235)	4.07 (9/221)	2.90 (7/241)	3.57 (7/196)	−0.301	0.802
DO Senior	1.53 (2/131)	1.18 (2/170)	1.56 (3/192)	1.94 (4/206)	1.20 (2/166)	−0.130	0.516
MD First	96.76 (179/185)	96.60 (227/235)	95.93 (212/221)	97.10 (234/241)	96.43 (189/196)	−0.333	0.972
MD Senior	98.47 (129/131)	98.82 (168/170)	98.44 (189/192)	98.06 (202/206)	98.80 (164/166)	0.412	0.146
Annals of EM							
DO First	4.22 (10/237)	2.43 (5/206)	3.80 (9/237)	2.10 (5/238)	1.88 (5/266)	0.208	0.248
DO Senior	1.99 (4/201)	0.66 (1/152)	1.78 (3/169)	1.17 (2/171)	0.93 (2/214)	−0.097	0.480
MD First	95.78 (227/237)	97.57 (201/206)	96.20 (228/237)	97.90 (233/238)	98.12 (261/266)	−0.136	0.522
MD Senior	98.01 (197/201)	99.34 (151/152)	98.22 (166/169)	98.83 (169/171)	99.07 (212/214)	0.390	0.156

*DO*, Doctor of Osteopathic Medicine; *MD*, Doctor of Medicine; *Annals, Annals of Emergency Medicine*; *AEM*, *Academic Emergency Medicine*; *JEM*, *Journal of Emergency Medicine*

**Table 2:** Percent representation of osteopathic physicians among first and senior authors of published articles in three journals of emergency medicine who held a dual degree.

	1995	2000	2005	2010	2014	Adj R Sq	P-value
Overall							
DO First	1.27 (1/79)	3.48 (4/115)	2.10 (3/143)	1.32 (3/227)	1.22 (3/246)	0.052	0.350
DO Senior	0 (0/80)	1.19 (1/84)	0.78 (1/128)	0 (0/161)	0.93 (2/214)	0.060	0.344
MD First	98.73 (78/79)	96.52 (111/115)	97.90 (140/143)	98.69 (224/227)	98.78 (243/246)	0.955	0.003
MD Senior	100 (80/80)	98.81 (83/84)	99.22 (127/128)	100 (161/161)	99.07 (212/214)	0.934	0.005
JEM							
DO First	0 (0/16)	5.56 (1/18)	0 (0/27)	3.64 (2/55)	0 (0/61)	−0.277	0.740
DO Senior	0 (0/25)	0 (0/15)	0 (0/22)	0 (0/50)	1.61 (1/62)	0.278	0.209
MD First	100 (16/16)	94.44 (17/18)	100 (27/27)	96.36 (53/55)	100 (61/61)	0.853	0.016
MD Senior	100 (25/25)	100 (15/15)	100 (22/22)	100 (50/50)	98.39 (61/62)	0.687	0.052
Acad EM							
DO First	0 (0/35)	4.35 (2/46)	3.57 (2/56)	1.34 (1/88)	2.53 (2/79)	0.041	0.358
DO Senior	0 (0/24)	2.86 (1/35)	1.79 (1/56)	0 (0/64)	1.31 (1/76)	−0.231	0.653
MD First	100 (35/35)	95.65 (44/46)	96.43 (54/56)	98.86 (87/88)	97.47 (77/79)	0.864	0.014
MD Senior	100 (24/24)	97.14 (34/35)	98.21 (55/56)	100 (64/64)	98.68 (75/76)	0.998	0.000
Annals of EM							
DO First	3.57 (1/28)	1.96 (1/51)	1.67 (1/60)	1.19 (1/84)	0.94 (1/106)	-	-
DO Senior	0 (0/31)	0 (0/34)	0 (0/50)	0 (0/47)	0 (0/76)	-	-
MD First	96.43 (27/28)	98.04 (50/51)	98.33 (59/60)	98.81 (83/84)	99.06 (105/106)	0.971	0.001
MD Senior	100 (31/31)	100 (34/34)	100 (50/50)	100 (47/47)	100 (76/76)	0.823	0.021

*DO*, Doctor of Osteopathic Medicine; *MD*, Doctor of Medicine; *Annals, Annals of Emergency Medicine*; *AEM*, *Academic Emergency Medicine*; *JEM*, *Journal of Emergency Medicine*
